# Increasing the adoption of home dialysis through improved advanced kidney care patient education: a call for action

**DOI:** 10.1093/ckj/sfaf087

**Published:** 2025-03-27

**Authors:** Ulrika Hahn Lundström, Gert Meeus, Tommy Aronsen, Anne-Lorraine Clause, Jeanette Finderup, Patrik J Finne, Jan Dominik Kampmann, Jacek Lange, Kate McCarthy, Rita Nohra, Tomasz Stompòr, Eleri Wood, Monika Lichodziejewska-Niemierko, Stefan H Jacobson

**Affiliations:** Division of Renal Medicine, Department of Clinical Science, Intervention and Technology, Karolinska Institutet, Stockholm, Sweden; Department of Nephrology, AZ Groeninge Hospital, Kortrijk, Belgium; Department of Medicine, Drammen Hospital, Drammen, Norway; Hôpital Erasme, HUB, Département de Néphrologie, dialyse et transplantation, Erasme, ULB, Brussels, Belgium; Department of Clinical Medicine, Aarhus University and Department of Renal Medicine Aarhus University Hospital, Aarhus, Denmark; Department of Nephrology, Helsinki University Hospital and University of Helsinki, Helsinki, Finland; Department of Internal Medicine, University Hospital of Southern Jutland, Sønderborg & Institute of Regional Health Research, University of Southern Denmark, Odense, Denmark; Baxter Poland, Warsaw, Poland; Baxter Healthcare, Compton, UK; Department of Medical Affairs, University of Uppsala, Uppsala, Sweden; Department of Nephrology, Hypertension and Internal Medicine, University of Warmia and Mazury, Olsztyn, Poland; Renal Department, King's College Hospital, London, UK; Department of Nephrology, Transplantology and Internal Medicine & Department of Palliative Medicine, Medical University of Gdańsk, Gdańsk, Poland; Division of Nephrology, Department of Clinical Sciences, Karolinska Institutet, Danderyd Hospital, Stockholm, Sweden

**Keywords:** patient education, guidelines, advanced kidney care, home dialysis, choice of treatment modality, haemodialysis, chronic renal failure, CKD, dialysis, peritoneal dialysis

## Abstract

**Background:**

Home dialysis modalities have several advantages yet remain underused in Europe. A minority of people with kidney failure opt for home dialysis, although many more could be suitable. To improve home dialysis uptake, advanced kidney care patient education is essential. The aim was to examine the association of national guidelines for advanced kidney care patient education with home dialysis prevalence and incidence across Europe.

**Methods:**

This call for action followed a consensus meeting in Copenhagen, Denmark, in June 2023. The participating professionals had extensive experience in advanced kidney care and home dialysis. We used data from the European Renal Association registry 2021 to examine the association of available national guidelines for advanced kidney care education with home dialysis prevalence and incidence in Europe.

**Results:**

In the European dialysis population, home dialysis prevalence is 10.5% and incidence is 13.3%. The organization of advanced kidney care and patient education differ. The availability of national guidelines for advanced kidney care patient education is associated with home dialysis uptake. The prevalence of home dialysis is significantly higher in countries with versus without national guidelines [20.9 versus 7.9%; odds ratio 1.398 (confidence interval 1.115–1.754), *P* = .004].

**Conclusion:**

Home dialysis prevalence and incidence vary in Europe. The availability of national guidelines for advanced kidney care patient education for professionals is associated with a higher prevalence and incidence of home dialysis. Coordinated action is needed to support advanced kidney care patient education as part of nephrology care to improve kidney care, in order to ensure that the right patient is on the right modality and increase access to home dialysis.

KEY LEARNING POINTS **What was known:**

- The uptake of home dialysis [peritoneal and home haemodialysis (HD)] in Europe remains low compared with in-centre HD, despite several acknowledged advantages and equal clinical outcomes.
- As already stated by a European Renal Best Practice advisory board >10 years ago, individualized advanced kidney patient care is vital to help persons with end-stage kidney disease make an informed decision about end-stage kidney care and the choice of dialysis modality.
- More than a decade later, guidelines for advanced kidney care patient education are still only available in a minority of European countries.

**This study adds:**

- This study demonstrated that the availability of national guidelines for advanced kidney care correlates significantly with a higher prevalence and 91-day incidence of home dialysis.
- In countries with a national guideline, the rate of home dialysis uptake is more than twice the rate seen in countries without a guideline, although in low-prevalence countries without national guidelines, individual centres may achieve a similar uptake through local guidelines.
- This study highlights the value of coordinated action to support advanced kidney care patient education to achieve more individualized kidney care and consequently improve home dialysis uptake.

**Potential impact:**

- A coordinated national policy with guidance for a structured approach to advanced kidney care patient education is a powerful tool to improve access to advanced kidney care patient education but is still insufficiently developed in many countries.
- Future nephrology care logistics and resources should be directed to support coordinated advanced kidney care patient education and should add ‘the right end-stage kidney disease treatment modality’ to the classic triad of ‘the right access at the right time for the right patient for the right reason’.
- Improved access to advanced kidney care patient education will pave the way for more individualized kidney care, with equal access to treatment, increased patient involvement in the choice of end stage kidney disease treatment modality and ultimately more patients opting for home dialysis.

## INTRODUCTION

Chronic kidney disease (CKD) affects >100 million people in Europe, 600 000 of whom are dependent on kidney replacement therapy (KRT) for their survival [1]. Access to the different forms of end-stage kidney disease (ESKD) care—dialysis, kidney transplantation and conservative care—varies in Europe [2]. The home dialysis modalities—peritoneal dialysis (PD) and home haemodialysis (HHD)—have several advantages but remain underused in many countries. Only a minority of European persons with ESKD opt for a home dialysis modality, although many more could be suitable for home dialysis [1, 3]. There is a strong association between advanced kidney care patient education and increased adoption of home dialysis, especially PD [4]. Likewise, nephrological follow-up in the year before dialysis initiation is associated not just with improved patient survival, but also with increased rates of kidney transplantation [5]. Validated prediction tools such as the Kidney Failure Risk Equation, could be further implemented to guide and prioritize decisions on patient care to predict short-term prognosis and timing of access referral and placement [6].

With the predicted future ‘silver tsunami’ of older and more frail persons with advanced CKD, both logistics and resources call for increased patient involvement in the choice of ESKD treatment and future goals of care [1, 7]. Therefore, individualized care for a person with ESKD is central to current nephrology guidelines [8]. This involves the right access at the right time for the right patient and for the right reason, hence the right dialysis modality is equally important [9].

In 2010, an appointed European Renal Best Practice, (ERBP) work group issued clinical advice on education of the person with ESKD in dialysis modality selection [10]. Nevertheless, there is still no consensus on the organization and availability of advanced kidney care patient education [11]. Available guidelines mainly focus on the multidisciplinary care of patients, although they offer few details on advanced kidney care patient education for CKD healthcare professionals [9, 12, 13]. A majority of European countries also lack national guidelines for advanced kidney care patient education [14]. In this article, we aim to study the correlation between the availability of national guidelines for advanced kidney care patient education for healthcare professionals and the adoption rates of home dialysis in European countries.

## MATERIALS AND METHODS

A consensus group of experienced European CKD home dialysis healthcare professionals was organized by Baxter Healthcare. Nine nephrologists and two registered nurses from 11 nephrology units were invited to participate. The represented nephrology units and the healthcare professionals were selected because of their years of experience working with advanced kidney care education and home dialysis, PD and HHD. The participants completed a questionnaire about the availability and content of predialysis education guidelines for advanced kidney care patient education in the participating countries. The questionnaire was designed by the workgroup of healthcare professionals together with Baxter Healthcare. The result of the questionnaire and how to proceed were discussed in several webinars during the spring, leading up to a meeting in Copenhagen in June 2023. At the meeting, experts split into four groups to discuss our purpose of developing recommendations for advanced kidney care patient education and pathways for persons with advanced kidney disease to promote home dialysis uptake.

We used data from the European Renal Association (ERA)-European Dialysis and Transplant Association (EDTA) registry to study the correlation of national guidelines for advanced kidney care with home dialysis prevalence and incidence by day 91 of kidney replacement therapy (KRT) in Europe [14]. In addition, data from Germany were collected from registries by the Kuratorium für Dialyse und Nierentransplantation (KfH). To calculate KRT prevalence and incidence by day 91, the number of patients using home dialysis was divided by the total number of dialysis patients. To study the presence of national guidelines for advanced kidney care education, a search for available national guidelines was performed and then confirmed by personal communication with healthcare professionals from each country.

### Statistical analysis

We analysed the reported data from the ERA Registry 2021 and KfH from 2022 [14] to calculate the prevalence and 91-day incidence of home dialysis, PD and HHD per country. We also calculated the mean values for prevalence and 91-day incidence of the same three dialysis modalities per country and group of countries with or without existing national guidelines. The two-sample *t*-test was used to calculate the mean prevalence and 91-day incidence per group of countries with or without an existing national guideline and logistic regression analysis was used to calculate the odds ratio (OR) for adoption of a home dialysis modality in countries with or without an existing national guideline.

## RESULTS

### Prevalence

**Figure 1:**
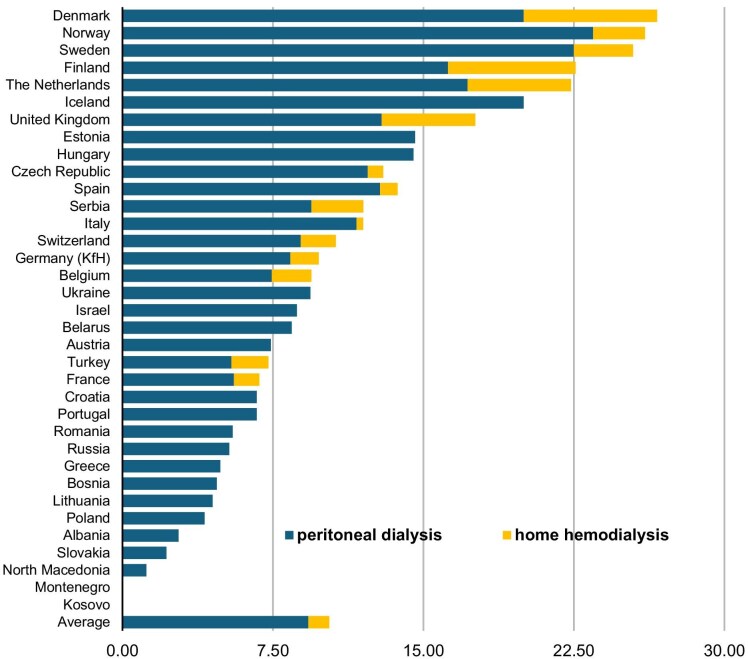
Home dialysis prevalence in Europe. Adapted from ERA-EDTA 2022 [17].

Prevalence data on home dialysis proportions were available from 35 European countries. The share of patients performing home dialysis in Europe is on average 10.5% (range 0.0–26.7). The prevalence of home dialysis is >20% in six European countries: Denmark, Norway, Sweden, Finland, The Netherlands and Iceland. The prevalence is <5% in nine countries: Greece, Bosnia, Lithuania, Poland, Albania, Slovakia, North Macedonia, Montenegro and Kosovo and <15% in 28 of the 35 European countries (Fig. 1).

### 91-day incidence

**Figure 2:**
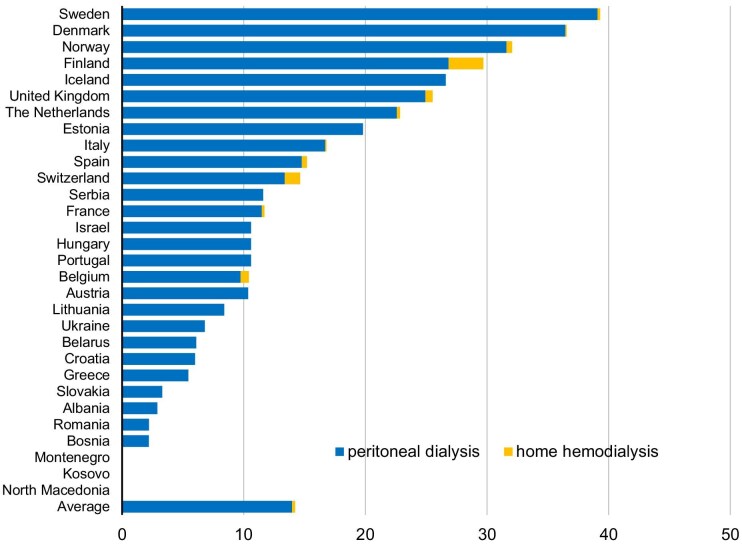
Home-dialysis incidence in Europe, day 91. Adapted from ERA-EDTA 2022 [17].

The 91-day incidence data on home dialysis proportions were available from 30 European countries*.* The incidence of home dialysis by day 91 is on average 13.3% (range 0.0–39.3) in Europe. The incidence is >20% in seven European countries: Sweden, Denmark, Norway, Finland, Iceland, the UK and The Netherlands. Incidence is reported at <5% in six countries—Slovakia, Albania, Romania, Bosnia, Montenegro and Kosovo—and <15% in 20 of the 30 countries that report incidence data on day 91 (Fig. 2).

### Availability of guidelines

National guidelines on advanced kidney care were available in 7 of 35 European countries (Table 1). In countries with national guidelines, the average home dialysis prevalence is 20.9% (range 6.7–26.7), compared with 7.9% (range 0–20.0) in the 28 countries without guidelines (*P* < .001). The OR for home dialysis prevalence in countries with national guidelines is 1.398 (confidence interval [CI] 1.115–1.754, *P* = .004) compared with countries without guidelines.

**Table 1:** Available European guidelines on CKD/home dialysis.

Denmark	National klinisk retningslinje for en behandlingsstrateguiden dialyse for patienter med kronisk nyresvigt. Dansk Nefrologisk selskat (DNS) og Faglig Selskab for Nefrologiske Sygeplejerster (FS Nefro) 2020
Norway	https://www.helsedirektoratet.no/statistikk/kvalitetsindikatorer/behandling-av-sykdom-og-overlevelse/andel-dialysepasienter-som-har-hjemmedialyse/Andel%20dialysepasienter%20som%20f%C3%A5r%20hjemmedialyse.pdf https://www.helsenorge.no/sykdom/nyrer-og-urinveier/nyresykdom-kronisk/
Sweden	https://ekpf.eu/wp-content/uploads/2020/07/ENG-Swedish_Translated_National-Care-program_20191206.pdf
Finland	https://bin.yhdistysavain.fi/1589922/WlXd6VuwLMwxn7cHIWjU0YoqPu/CKD-strategy_english.pdf
The Netherlands	Federatie Medisch Specialisten. Chronic kidney damage [Chronische nierschade (CNS)]. Utrecht. https://richtlijnendatabase.nl/richtlijn/chronische_nierschade_cns/startpagina_-_chronische_nierschade_cns.html.
UK	National Institute for Health and Care Excellence, Recommendations for information and education for people with CKD: guideline NG203 (2021); National Institute for Health and Care Excellence, Recommendations for CKD5 education: guideline NG107 (2018)
Portugal	Chronic kidney disease: a practical guide (ACT-NAU) Portuguese journal of family medicine and general, 2024; vol 40, supplement 11 https://rpmgf.pt/ojs/index.php/rpmgf/article/view/13985/11919

Portugal is the only one of 21 countries with national guidelines that has a home dialysis prevalence <10%. The average home dialysis incidence is 28.1% (range 18.5–37.7) in countries with national guidelines (*n* = 7) compared with 8.9% (range 2.19–15.5) in countries without national guidelines (*n* = 23) (*P* < .001). The OR for home dialysis incidence by day 91 in countries with available national guidelines is 1.276 (CI 1.070–1.522, *P* = .007) compared with countries without guidelines (Table 2).

**Table 2:** Home dialysis prevalence and incidence at day 91 by availability of national guidelines by country.

Country	Home dialysis prevalence (%)	Home dialysis incidence by day 91 (%)	National guidelines
Denmark	26.7	36.6	Yes
Norway	26.1	32.1	Yes
Sweden	24.5	39.3	Yes
Finland	22.6	29.7	Yes
The Netherlands	22.4	22.9	Yes
Iceland	20.0	26.6	No
UK	17.6	25.5	Yes
Estonia	14.6	19.8	No
Hungary	14.5	10.6	No
Czech Republic	14.0		No
Spain	13.7	15.2	No
Serbia	12.0	11.6	No
Italy	12.0	16.8	No
Switzerland	10.6	14.6	No
Germany (KfH)	9.8		No
Belgium	9.4	10.4	No
Ukraine	9.4	6.8	No
Israel	8.7	10.8	No
Belarus	8.4	6.1	No
Austria	7.4	10.7	No
Turkey	7.3		No
France	6.8	11.5	No
Croatia	6.7	6.0	No
Portugal	6.7	10.6	Yes
Romania	5.5	2.2	No
Russia	5.3		No
Greece	4.9	5.5	No
Bosnia	4.7	2.2	No
Lithuania	4.5	8.4	No
Poland	4.1		No
Albania	2.8	2.9	No
Slovakia	2.2	3.3	No
North Macedonia	1.2		No
Montenegro	0	0	No
Kosovo	0	0	No

## DISCUSSION

We found notable differences in the prevalence and incidence of home dialysis between European countries. The proportion of home dialysis at the end of 2021 was >25% in two countries and all countries with a home dialysis prevalence >20% have established national guidelines for the education of advanced kidney care professionals. Conversely, no country with <15% home dialysis prevalence, except for Portugal, has national guidelines for advanced kidney patient care and education [12]. Portugal is the only country of the 21 countries with <10% home dialysis despite the presence of national guidelines [15]. There are several explanations for the variable home dialysis uptake, and we can only speculate on the reasons for low uptake of which lack of guidelines may be one reason. In the literature, differences in regulations and resources, lack of available treatment options or issues to support home dialysis have been mentioned [16]. Furthermore, funding and reimbursement issues, such as the number of nephrologists per country, not enough staff or hospital resources and training facilities may play a role. Issues such as the attitude of both healthcare professionals and persons with CKD toward home dialysis, fear of needles or invasive procedures without professional staff around, aversion to medicalize one’s home, no fixed home address or just personal preference may also contribute to differences in uptake [16, 18, 19].

Incidence data per day 91 on home dialysis rates are available from 30 European countries.

The share of patients performing home dialysis in Europe is on average 13.3%. The incidence is >20% in seven European countries and home dialysis incidence is <10% in 12 countries: Lithuania, Ukraine, Belarus, Croatia, Greece, Slovakia, Albania, Romania, Bosnia, Montenegro, Kosovo and North Macedonia. In 20 countries the incidence is greater than the prevalence, possibly due to an increasing trend for home dialysis, failure of home dialysis after the initial 3 months or a high renal transplant rate. Conversely, in seven countries the prevalence is greater than the incidence, possibly reflecting a decreasing trend for home dialysis, superior survival of patients on home dialysis compared with in-centre HD or lower transplant rates.

The availability of guidelines for advanced kidney care varies substantially in Europe, although previous studies have shown that recognition of CKD within national health policies is critical to improving kidney healthcare [20]. National guidelines were available in six of the seven countries with the highest home dialysis prevalence. In countries where no national guidelines for advanced kidney care patient education exist, adoption of home dialysis is generally lower than in countries with guidelines. Portugal is an exception. Personal communication with a Portuguese nephrologist confirmed there is no statement about home dialysis in the guidelines or national good practices manual.

Several reasons may influence the presence of national guidelines for home therapies or for addressing CKD-specific policies. The existence of CKD policies varied according to country income. Low- and lower-middle-income countries were less likely to have policies addressing CKD or have governments recognizing it as a health priority. National CKD policies and advocacy organizations for CKD were more often seen in upper-middle and high-income countries. Studies have shown that while international clinical guidelines provide recommendations about best practices, country-specific national guidelines play an important role in interpreting the recommendations in the context of national prevalence patterns and resource availability [20].

Implementation of regional education guidelines can improve uptake in countries with a lower home dialysis rate on the national level [21]. For instance, a Polish centre reported a 30% peritoneal dialysis incidence despite a country prevalence of 4.1% [22]. Therefore, regulatory, sociocultural and logistic barriers can be overcome through dedicated advanced kidney care education for healthcare professionals.

Advanced kidney care patient education is a prerequisite for informed shared decision-making, to align the needs and preferences of the person with their preferred ESKD treatment modality [23]. Hence, continual and recurrent kidney care patient education is important at several stages during CKD [23, 24]. First, at the nephrology referral, to slow the disease progression rate. Then, in the predialysis stage, including patients with failing kidney transplants, for selection of the most appropriate dialysis modality, pretransplant investigation and dialysis access planning. Then, for patients dialyzing at in-centre HD facilities, to promote self-care, home dialysis transfer and facilitation of life participation [25]. Finally, to support the person for whom conservative supportive care is an option [26].

There are several examples of advanced kidney care education programs for professionals (Table 2). The educational framework can provide an individualized, holistic evaluation of the capabilities, needs and preferences of the person with ESKD. Increased knowledge and empowerment foster the shared decision-making process and increase each patient’s confidence in self-care and home dialysis. Ideally a structured advanced kidney care education program would identify the educational needs of each person with ESKD to solve any potential barriers to the choice of home dialysis [24].

In advanced kidney care education programs for healthcare professionals involved in advanced kidney care, a widespread, uniform approach is needed to further improve uptake in home dialysis. Delivering individualized advanced kidney care information on all treatment options requires skills and training for healthcare professionals [11]. Even in countries with national guidelines in place, a more structured standardized advanced kidney care education program could improve education quality and home dialysis uptake [27]. In studies, persons with kidney disease request unbiased individual counselling rather than education alone [18]. It is time to learn from the different best practices and experiences to develop a structured framework for advanced kidney care education, with quality standards to ensure high-quality education aimed at helping patients make informed choices regarding the most suitable dialysis modality [28]. There are also interesting initiatives to learn from, including virtual staff training programs to provide qualitative education [29].

### Strengths and limitations

Some European countries report incomplete data on dialysis modality to the ERA registry. Data from Italy and Germany were incomplete but were nevertheless included since they represent a large number of patients. Several factors may impact differences in practice patterns and variations in home dialysis uptake, such as funding, availability of certain treatment modalities and accessibility to kidney transplantation, as well as socio-economic and cultural differences, the adoption of conservative care and the possibility of assisted PD, to mention only a few.

## CONCLUSION

In this study on home dialysis uptake in Europe, the home dialysis prevalence and incidence by day 91 vary considerably between European countries. We found that the availability of national guidelines for advanced kidney care patient education for healthcare professionals is associated with home dialysis prevalence and incidence. In countries with available national guidelines for advanced kidney care education, both the prevalence and incidence of home dialysis were higher. Coordinated action is needed to establish an advanced kidney care patient education program for healthcare professionals as an essential part of comprehensive CKD patient care. This would help to ensure that the right patient is on the right modality according to their individualized needs and increase access to home dialysis for more patients.

## Supplementary Material

sfaf087_Supplemental_File

## Data Availability

No new data were generated or analysed in support of this research.
