# Supplementary material for: Increasing the adoption of home dialysis through improved advanced kidney care patient education: a call for action
Source: Clin Kidney J. 2025 Mar 27;18(4):sfaf087. doi: 10.1093/ckj/sfaf087 (PMC11986811; doi:10.1093/ckj/sfaf087)
Supplement: sfaf087_Supplemental_File [file sfaf087_supplemental_file.docx]

Supplement 1 : Questions to the working group

| How does the patient education program look like at your hospital? |
| --- |
| How does the organization at your hospital look like? |
| Do you have different programs for different CKD stages? |
| Are you aware of any other programs from other regions/ hospitals in your country? |
| Is there a consensus on a national predialysis education program in your country? |
